# 531. Comparative Efficacy of Pharmacological Treatments for Burn-induced Hypermetabolism: A Systematic Review and Network Meta-analysis

**DOI:** 10.1093/jbcr/irag033.228

**Published:** 2026-04-06

**Authors:** Cameron J Leong, Noah Ballinger, Brandon Chai, Anthony Papp

**Affiliations:** The University of British Columbia, Vancouver, BC; The University of British Columbia, Victoria, BC; The University of British Columbia, Vancouver, BC; Vancouver General Hospital BC Burn Unit, Vancouver, BC

## Abstract

**Introduction:**

Patients with burns covering >15% of their total body surface area (TBSA) are susceptible to developing a hypermetabolic response characterized by accelerated catabolism and increased resting energy expenditure. This response is also associated with insulin resistance and hyperglycemia, resulting in impaired wound healing, increased risk of infection, multi-organ dysfunction, and sometimes death. While non-pharmacological treatments, such as early excision and enteral nutrition, are essential, pharmacological interventions, including beta-blockers, anabolic agents, and insulin sensitizers, are used with varying efficacy. This study aims to compare the various pharmacological treatments for hypermetabolism.

**Methods:**

In accordance with PRISMA guidelines, a systematic literature review in Ovid MEDLINE, EMBASE, Scopus, Cochrane Central Register of Controlled Trials, and CINAHL was conducted from Jan 1st, 2000- May 1st, 2025. Studies of adult patients with major burns (>15% TBSA) receiving pharmacological treatment for hypermetabolism were included. Odds ratios and mean differences were estimated using network meta-analysis with random effects.

**Results:**

Out of 1534 studies found, 25 studies including 12 585 burn patients were included in the analysis. Oxandrolone (OR = 0.30, 95% CI = 0.11-0.83) and testosterone (OR 0.07, 95% CI = 0.01-0.69) had a mortality benefit compared to control (placebo, no treatment, or non-intensive insulin). When adjusting for differences in %TBSA and for differences in inhalation injury between treatment arms, metformin also had a mortality benefit (OR = 0.21, 95% CI = 0.05-0.95). Combination therapy of beta-blockers and oxandrolone increased average length of hospitalization (MD = 14.46, 95% CI = 1.56-27.37). However, this result was not significant after adjusting for differences in mean age or differences in inhalation injury.

**Conclusions:**

Oxandrolone and testosterone have a mortality benefit compared to control for the treatment of burn-induced hypermetabolism. Metformin may also have a mortality benefit when adjusting for differences in TBSA. There is no evidence for the use of other pharmacological treatments for reducing mortality or duration of hospitalization.

**Applicability of Research to Practice:**

This study will help clinicians select the most effective pharmacological strategies to improve outcomes in adult burn patients with hypermetabolism.

**Funding for the study:**

N/A.

